# The effect of exercise on flow-mediated dilation in people with type 2 diabetes mellitus: a systematic review and meta-analysis of randomized controlled trials

**DOI:** 10.3389/fendo.2024.1347399

**Published:** 2024-03-26

**Authors:** Bopeng Qiu, Yilun Zhou, Xifeng Tao, Xiao Hou, Liwen Du, Yuanyuan Lv, Laikang Yu

**Affiliations:** ^1^ Department of Strength and Conditioning Assessment and Monitoring, Beijing Sport University, Beijing, China; ^2^ School of Physical Education, Xihua University, Chengdu, China; ^3^ School of Sport Sciences, Beijing Sport University, Beijing, China; ^4^ China Institute of Sport and Health Science, Beijing Sport University, Beijing, China

**Keywords:** exercise, endothelial function, flow-mediated dilation, type 2 diabetes mellitus, systematic review, meta-analysis

## Abstract

**Introduction:**

An increasing number of studies have investigated the effect of exercise on flow-mediated dilation (FMD) in people with type 2 diabetes mellitus (T2DM), while the findings were controversial. The primary aim of this systematic review and meta-analysis was to investigate the effect of exercise on FMD in T2DM patients, and the secondary aim was to investigate the optimal type, frequency, session duration, and weekly time of exercise for T2DM patients.

**Methods:**

Searches were conducted in PubMed, Cochrane Library, Scopus, Web of Science, Embase and EBSCO databases. The Cochrane risk of bias tool (RoB2) in randomized trial and Physiotherapy Evidence Database (PEDro) scale were used to assess the methodological quality of the included studies.

**Results:**

From the 3636 search records initially retrieved, 13 studies met the inclusion criteria. Our meta-analysis revealed that exercise had a significant effect on improving FMD in T2DM patients [WMD, 2.18 (95% CI, 1.78-2.58), *p* < 0.00001, *I*^2^ = 38%], with high-intensity interval training (HIIT) being the most effective intervention type [HIIT, 2.62 (1.42-3.82); *p* < 0.0001; aerobic exercise, 2.20 (1.29-3.11), *p* < 0.00001; resistance exercise, 1.91 (0.01-3.82), *p* = 0.05; multicomponent training, 1.49 (0.15-2.83), *p* = 0.03]. In addition, a higher frequency [> 3 times, 3.06 (1.94-4.19), *p* < 0.00001; ≤ 3 times, 2.02 (1.59-2.45), *p* < 0.00001], a shorter session duration [< 60 min, 3.39 (2.07-4.71), *p* < 0.00001; ≥ 60 min, 1.86 (1.32-2.40), *p* < 0.00001], and a shorter weekly time [≤ 180 min, 2.40 (1.63-3.17), *p* < 0.00001; > 180 min, 2.11 (0.82-3.40), *p* = 0.001] were associated with larger improvements in FMD.

**Conclusion:**

This meta-analysis provides clinicians with evidence to recommended that T2DM patients participate in exercise, especially HIIT, more than 3 times per week for less than 60 min, with a target of 180 min per week being reached by increasing the frequency of exercise.

**Systematic review registration:**

https://www.crd.york.ac.uk/prospero, identifier CRD42023466575.

## 1 Introduction

Type 2 diabetes mellitus (T2DM) is a prevalent chronic metabolic disease usually due to defective insulin secretion from pancreatic β-cells and a blunted insulin response in insulin-sensitive tissues (1). Patients with T2DM exhibit hyperglycemia, excessive release of free fatty acids (FFAs), insulin resistance, and hyperinsulinemia (2). Endothelial dysfunction, one of the pathological features of T2DM (3), is usually defined as decreased nitric oxide (NO) bioavailability (4), which may be triggered by elevated oxidative stress, leading to increased reactive oxidative substances (ROS), thereby impairing vascular endothelial function (4, 5). In addition, endothelial dysfunction and atherosclerosis are important factors affecting vascular complications in T2DM patients (6).

Endothelial dysfunction leads to a significantly increased risk of chronic diseases such as cardiovascular diseases (CVDs) and its associated complications (7, 8), which is a major cause of morbidity and mortality in T2DM patients (9). A previous study showed that T2DM patients had a doubled risk of developing CVDs (10). Flow-mediated dilation (FMD) is the non-invasive gold standard method for assessing arterial endothelial function (11, 12). Several studies have demonstrated that brachial artery FMD serves as an independent predictor of cardiovascular events (13–15). Therefore, it is important to develop programs to improve endothelial function for the prevention and treatment of T2DM and its associated chronic diseases (16).

Exercise, diet, and medication are important tools in the treatment of T2DM (17). However, exercise interventions are more cost-effective and convenient than other interventions. Studies have shown that exercise can improve the health of T2DM patients, including cardiovascular function (18–20), inflammation (21), cognitive function, and metabolic health (22). A meta-analysis showed that exercise had a significant effect on FMD in different populations (23). With the consensus on exercise as a treatment for T2DM (17), the potential benefits of exercise on FMD in T2DM patients have attracted considerable attention (24–36). However, the type of intervention can have a different impact on FMD in T2DM patients. Of these, aerobic exercise is the most studied type of exercise for T2DM and usually involves exercises that mobilize whole-body muscle groups, such as running, swimming, and brisk walking (37). In addition, traditional aerobic exercise tends to use lower intensity exercise, which means that a longer duration may be required to achieve the corresponding exercise effect. Unfortunately, obesity is a common complication of T2DM, with 80% of T2DM patients having obesity (38). Due to limited mobility and peripheral neuropathy (39), it may be difficult for these patients to ensure good compliance when performing prolonged whole-body exercise (40). In such cases, using resistance exercise that stimulates localized muscle groups or using shorter high-intensity interval training (HIIT) sessions may be a better option (24, 40). However, the effect of exercise and other modalities on the efficacy of FMD in T2DM patients remains unclear.

Therefore, the primary aim of this systematic review and meta-analysis was to investigate the effect of exercise on FMD in T2DM patients, and the secondary aim was to investigate the optimal type, frequency, session duration, and weekly time of exercise for T2DM patients. We hypothesized that exercise would significantly improve FMD in T2DM patients, with HIIT being the most effective type of intervention, and that the frequency and session duration would influence the efficacy of the exercise intervention, with the optimal combination being a higher frequency (more than 3 times per week) and a shorter session duration (less than 60 min).

## 2 Methods

This study followed the Preferred Reporting Items for Systematic Reviews and Meta-Analyses guidelines (PRISMA, 2020) (41) and was registered on PROSPERO (CRD42023466575).

### 2.1 Search strategy

We searched the PubMed, Web of Science, Embase, EBSCO, Scopus, and Cochrane library for randomized controlled trials (RCTs) relating to the effect of exercise on endothelial function in T2DM patients from the inception dates to 20 February, 2024 (**Supplementary Table 1**). Reference lists of relevant studies, including reviews and meta-analyses, were manually searched to identify additional relevant studies. The procedure was performed independently by two authors (BQ and YZ), and disagreement were resolved through discussion with the third author (LY).

### 2.2 Eligibility criteria

Inclusion criteria were: (1) RCTs; (2) using T2DM patients as subjects; (3) including an intervention and control groups; (4) using FMD as the outcome measure and the data were present as percentage.

Exclusion criteria were: (1) non-English articles; (2) conference abstracts; (3) animal studies; (4) reviews.

### 2.3 Data extraction

The data extraction was conducted by two authors (BQ and YZ), including: (1) surname of the first author, publication year, and sample size; (2) categorized variable: intervention type [aerobic exercise, HIIT, resistance exercise, and multicomponent training (a training modality that involves different physical capacities in the same exercise session) (42)] and continuous variables: duration, session duration, frequency, and weekly time; (3) participants’ age and disease duration; and (4) mean and standard deviation (SD) values reflecting changes in FMD, as described previously (43).

### 2.4 Methodological quality assessment

The version 2 of the Cochrane risk of bias tool (RoB2) in randomized trial and Physiotherapy Evidence Database (PEDro) scale were used to assess the methodological quality of included studies (44, 45). RoB2 was assessed mainly from 7 items: random sequence generation (selection bias), allocation concealment (selection bias), blinding of participants and personnel (performance bias), blinding of outcome assessment (detection bias), incomplete outcome data (attrition bias), selective reporting (reporting bias), and other biases. For PEDro scale, 11 items were evaluated, where studies scoring < 4 points, 4-5 points, 6-8 points, and > 9 points are considered poor, average, good, and excellent quality, respectively (46).

### 2.5 Statistical analysis

Weighted mean differences (WMDs) and 95% confidence intervals (CIs) were used to estimate the effects of exercise on FMD in T2DM patients. For included studies reporting standard error (SE) or 95% CI, SD was calculated using the previously described formula (47). Heterogeneity was assessed using the *I* ^2^ static, where *I* ^2^ < 25% indicate no significant heterogeneity, 25% < *I* ^2^ < 50% indicate low heterogeneity, 50% < *I* ^2^ < 75% indicate moderate heterogeneity, and *I* ^2^ > 75% indicate high heterogeneity (48). Data were pooled using fixed effects models or random effects models when *I* ^2^ < 50% or *I* ^2^ ≥ 50%, respectively (49). Subgroup analysis, meta-regression analysis, and sensitivity analysis were used to interpret the results if there was a high heterogeneity (*I* ^2^ > 60%) (43).

For subgroup analyses, we examined the effect of intervention type (aerobic exercise, HIIT, resistance exercise, and multicomponent exercise), frequency (≤ 3 times and > 3 times), session duration (< 60 min and ≥ 60 min), and weekly time (≤ 180 min and > 180 min) on FMD in T2DM patients. Meta-regressions were conducted based on the participants’ age, disease duration, frequency, session duration, and weekly time. The forest plots were generated using Review manager software (Version 5.4; Cochrane Collaboration), and sensitivity analysis, meta-regressions, and funnel plot were performed using Stata software (Version 15.0, Stata Corp, College Station, Texas). Statistical significance was considered for outcomes with a *p* < 0.05.

## 3 Results

### 3.1 Study selection

**Figure 1:**
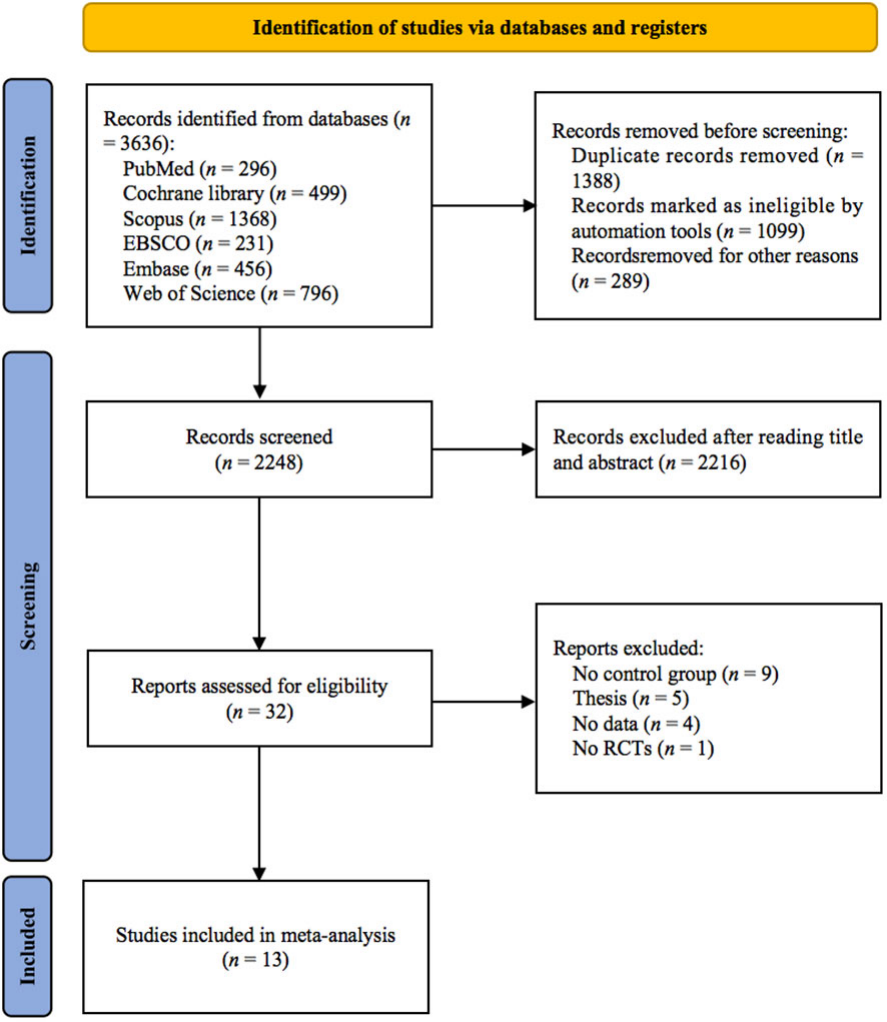
PRISMA flowchart of study selection.

As shown in **Figure 1**, 3636 studies were identified from 6 databases. After excluding duplicates, 2248 studies remained, and after screening titles and abstracts, 32 studies remained. Nineteen studies were excluded for the following reasons: (1) no control group (*n* = 9); (2) thesis (*n* = 5); (3) no data (*n* = 4); and (4) no RCTs (*n* = 1). Finally, 13 studies (24–36) met the inclusion criteria.

### 3.2 Study characteristics

As shown in **Supplementary Table 2**, among the included studies, there were 290 T2DM patients in the 18 intervention groups and 233 T2DM patients in the 13 control groups. Among the included studies, 3 studies (24, 30, 34) involved only women, 1 study (29) involved only men, and 9 studies (25–28, 31–35) involved both men and women. The mean age of the participants ranged from 15.3 to 70.5 years. The mean age of participants in 2 studies (24, 32) was < 45 years, and 11 studies (25–31, 33–36) involved participants with mean age ≥ 45 years. The mean time from T2DM to intervention of participants ranged from 1.43 to 21.1 years. Most interventions specified aerobic exercise (*n* = 7) (26, 28–31, 34, 35), high-intensity interval training (HIIT, *n* = 5) (24, 26–28, 31), resistance exercise (*n* = 3) (27, 30, 36), and multicomponent training (*n* = 3) (25, 32, 33). For aerobic exercise, the total duration of intervention ranged from 8 to 12 weeks, with an average of 11.3 weeks, the frequency of intervention per week was 3 times, and minutes of intervention per session ranged from 30 to 62 minutes, with an average of 54 minutes. For HIIT, the total duration of intervention was 12 weeks, the frequency of intervention per week was 3 times, the number of intervals ranged from 3 to 11 times, with an average of 7 times, the interval time ranged from 1 to 3 minutes, with an average of 2 minutes, and the number of repetitions per session ranged from 4 to 12 times, with an average of 8 times. For resistance exercise, the total duration of intervention was 12 weeks and the frequency of intervention per week was 3 times. For multicomponent training, the total duration of intervention ranged from 12 to 24 weeks, with an average of 16 weeks, the frequency of intervention per week ranged from 3 to 5 times, and minutes of intervention per session ranged from 60 to 75 minutes, with an average of 67.5 minutes. The session duration ranged from 19 to 75 min, while 1 study (36) did not provide information on session duration. The frequency ranged from 3 to 5 times per week, and we calculated the weekly time based on frequency and session duration (42), which ranged from 90 to 300 min. The results of FMD in all included studies were presented as percentages.

### 3.3 Risk of bias

**Figure 2:**
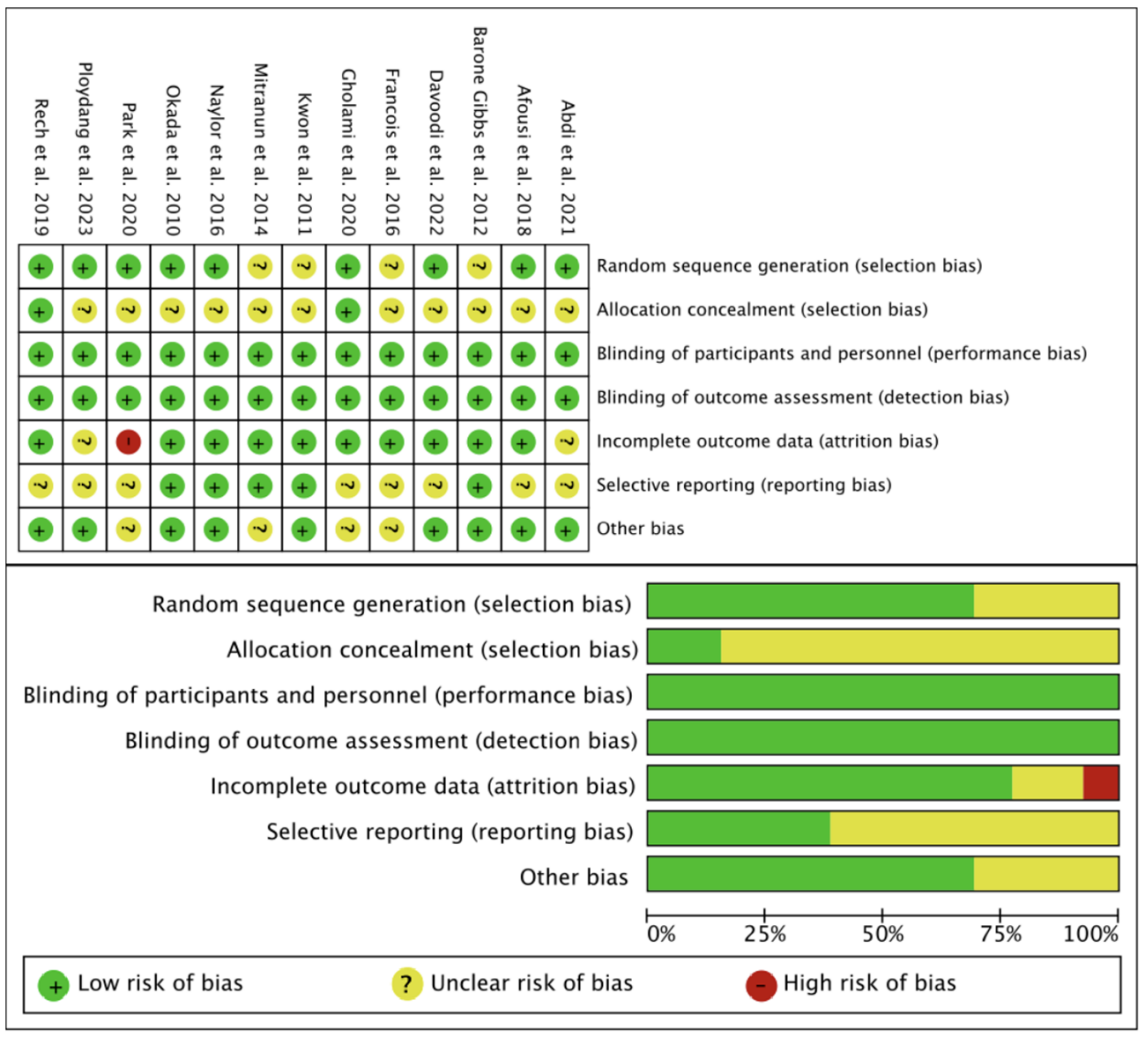
Results of Cochrane risk of bias tool.

The RoB2 was used to assess the quality of the included studies in terms of selection bias, performance bias, detection bias, attrition bias, reporting bias, and other bias (**Figure 2**). The PEDro scale showed that of the 13 included studies, 1 were of excellent quality and 12 was of good quality (**Supplementary Table 3**).

### 3.4 Meta-analysis results

#### 3.4.1 Effects of exercise on FMD in T2DM patients

**Figure 3:**
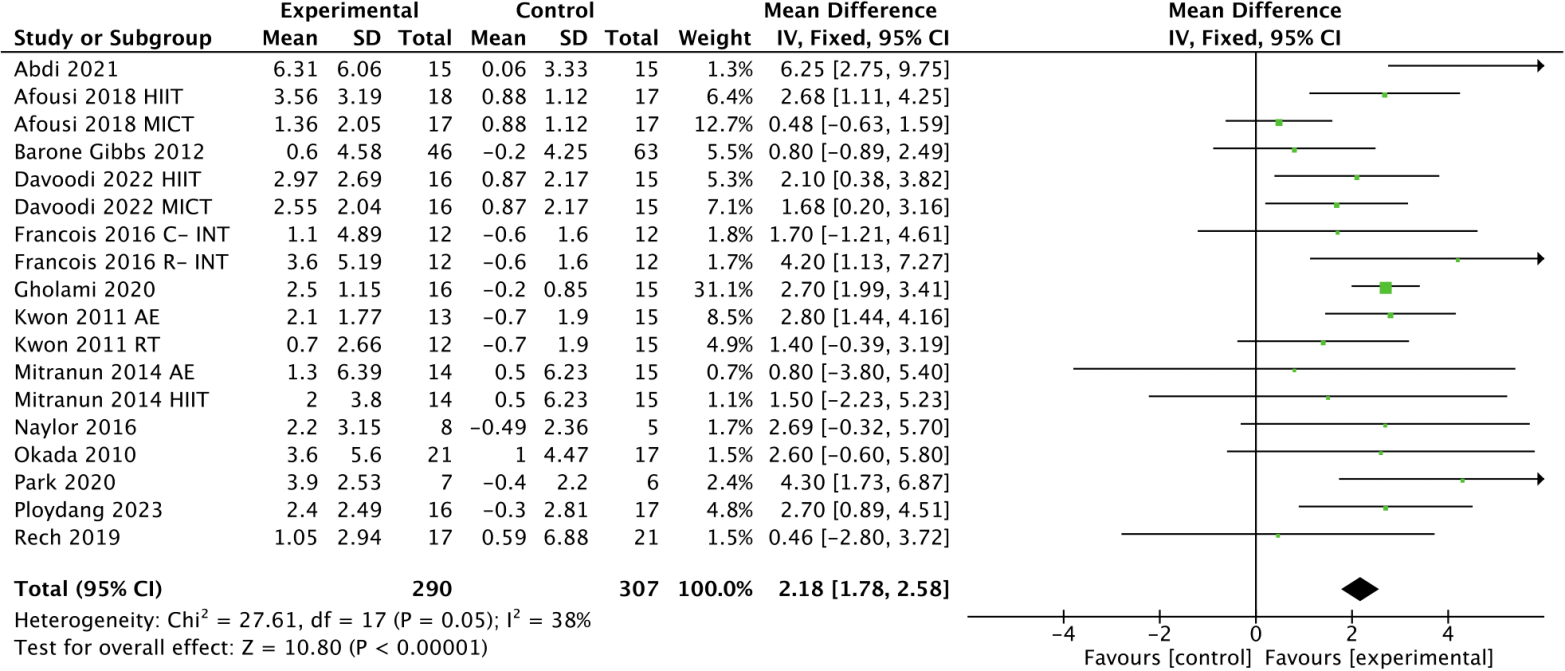
Meta-analysis results of the effect of exercise on FMD in T2DM patients.

Exercise had a significant effect on improving FMD in T2DM patients [WMD, 2.18 (95% CI, 1.78-2.58); *p* < 0.00001; *I* ^2^ = 38%; **Figure 3**].

#### 3.4.2 Subgroup analysis

**Figure 4:**
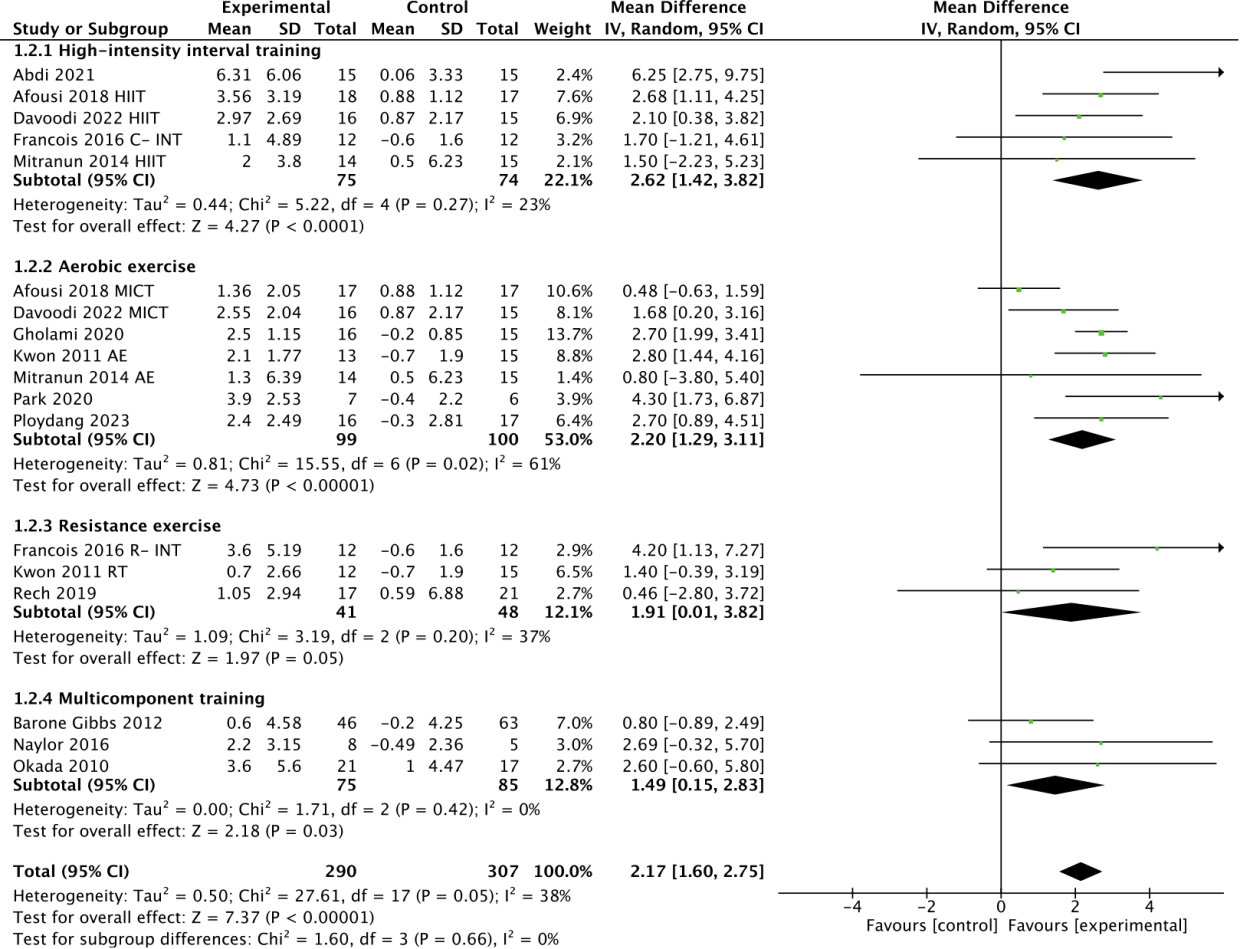
Meta-analysis results of the effect of different types of intervention on FMD in T2DM patients.

As shown in **Figure 4**, subgroup analysis showed that HIIT [WMD, 2.62 (95% CI, 1.42-3.82; *p* < 0.0001; *I* ^2^ = 23%], aerobic exercise [WMD, 2.20 (95% CI, 1.29-3.11); *p* < 0.00001; *I* ^2^ = 61%], resistance exercise [WMD, 1.91 (95% CI, 0.01-3.82); *p* = 0.05; *I* ^2^ = 37%], and multicomponent training [WMD, 1.49 (95% CI, 0.15-2.83); *p* = 0.03; *I* ^2^ = 0%] were effective in improving FMD in T2DM patients, with HIIT being the most effective intervention type.

**Figure 5:**
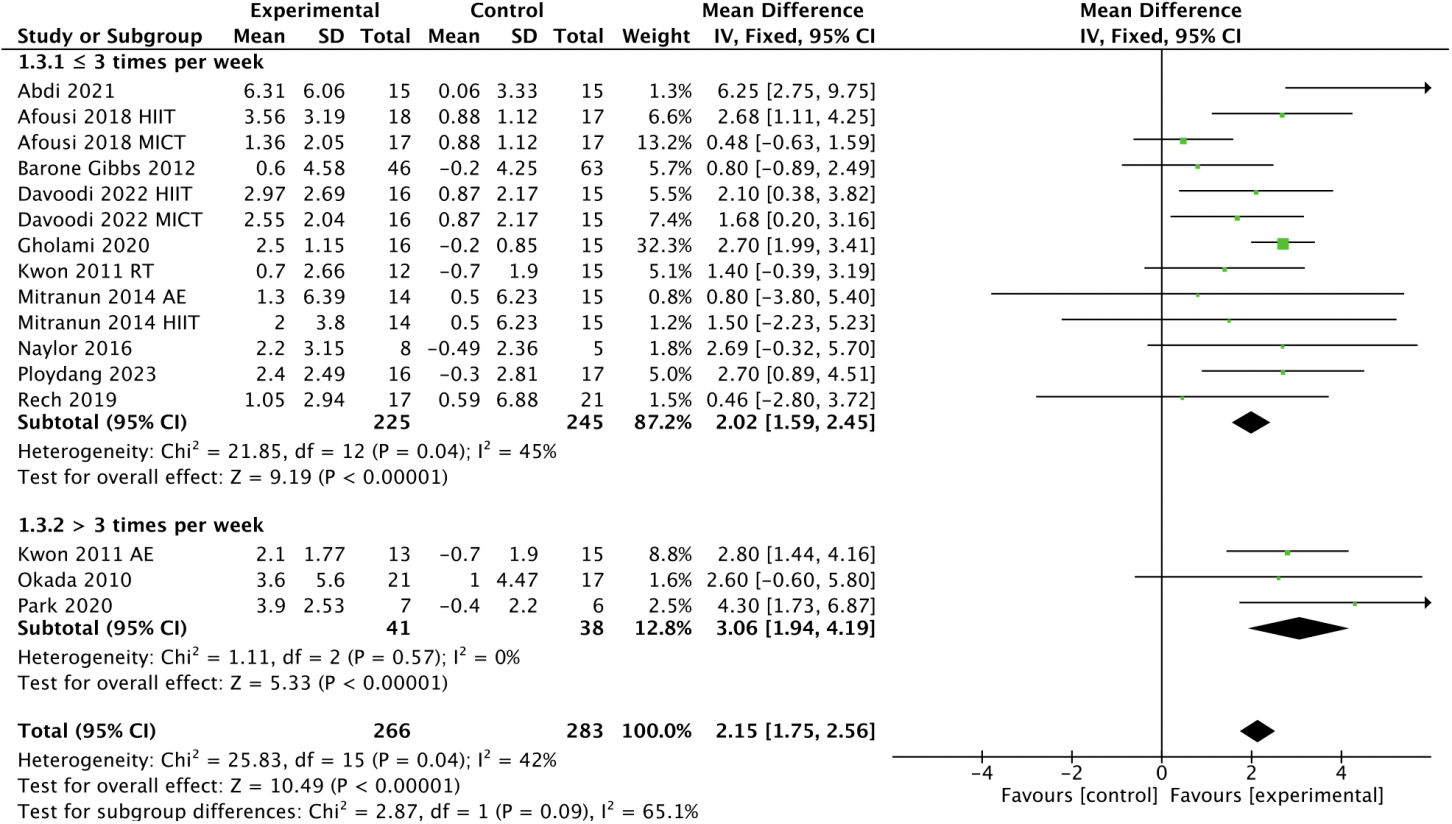
Meta-analysis results of the effect of the frequency of intervention on FMD in T2DM patients.

**Figure 6:**
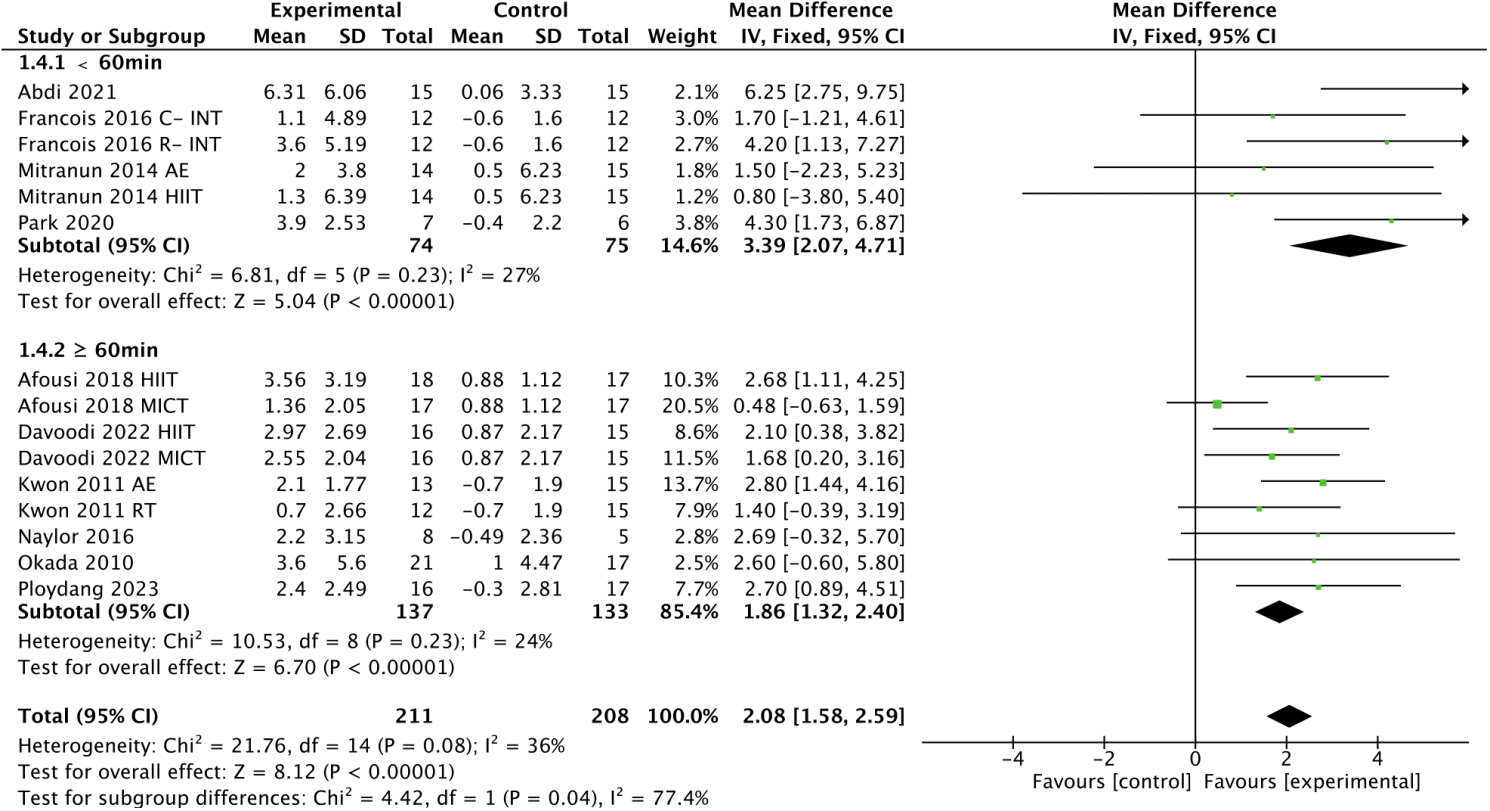
Meta-analysis results of the effect of the duration of intervention per session on FMD in T2DM patients.

**Figure 7:**
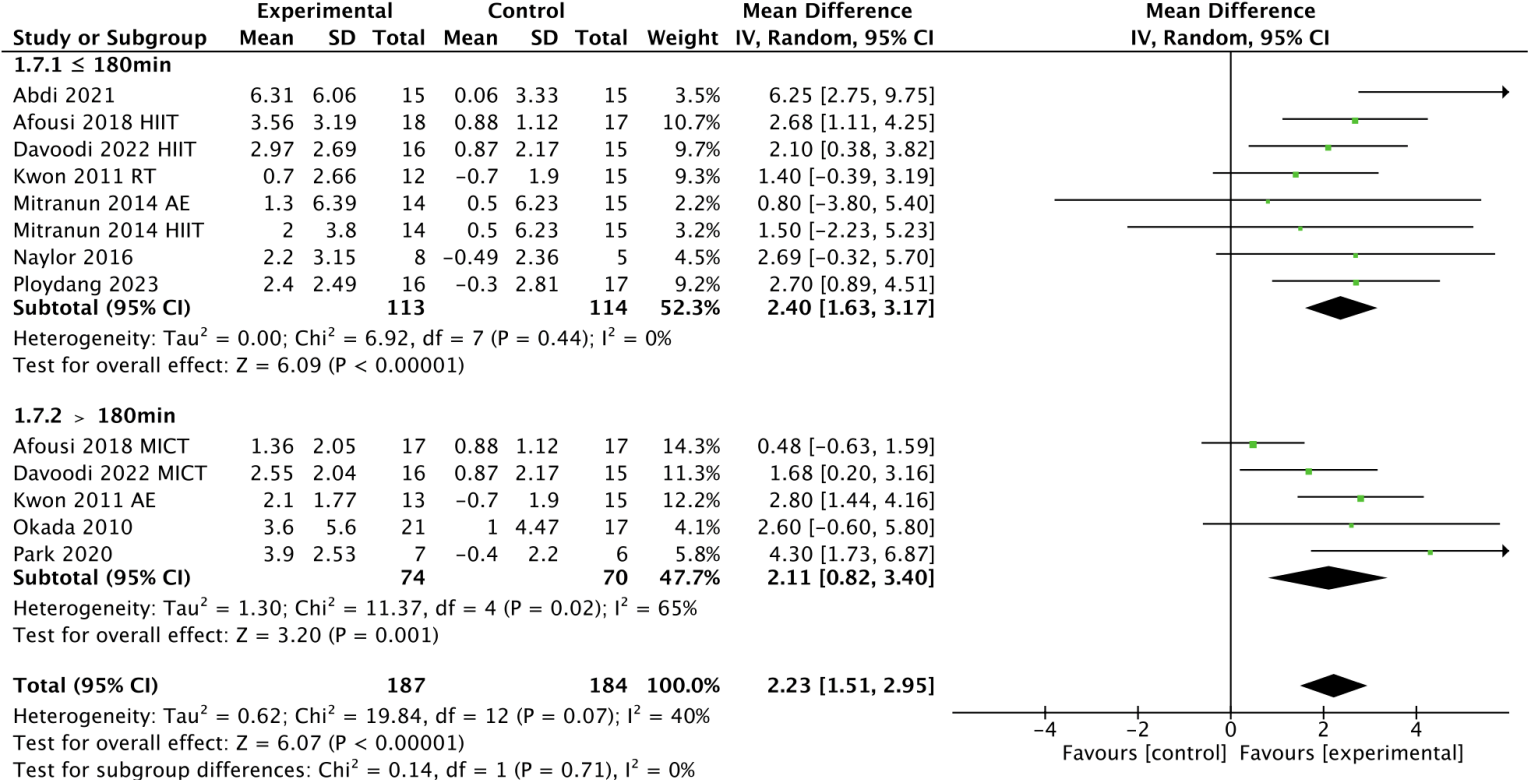
Meta-analysis results of the effect of the duration of intervention per week on FMD in T2DM patients.

In addition, subgroup analyses indicated that a higher frequency [> 3 times, WMD, 3.06 (95% CI, 1.94-4.19); *p* < 0.00001; *I* ^2^ = 0%; ≤ 3 times, WMD, 2.02 (95% CI, 1.59-2.45); *p* < 0.00001; *I* ^2^ = 45%; **Figure 5**], a shorter session duration [< 60 min, WMD, 3.39 (95% CI, 2.07-4.71); *p* < 0.00001; *I* ^2^ = 27%; ≥ 60 min, WMD, 1.86 (95% CI, 1.32-2.40); *p* < 0.00001; *I* ^2^ = 24%; **Figure 6**], and a shorter weekly time (≤ 180 min, WMD, 2.40 [95% CI, 1.63-3.17); *p* < 0.00001; *I* ^2^ = 0%; > 180 min, WMD, 2.11 (95% CI, 0.82-3.40); *p* = 0.001; *I* ^2^ = 65%; **Figure 7**] were associated with larger improvements in FMD.

### 3.5 Meta regression

Meta-regression analyses were performed on intervention duration, session duration, frequency, weekly time, participants’ age, and disease duration. No significant associations were observed between intervention duration (*p* = 0.128, **Supplementary Figure 1**), frequency (*p* = 0.144, **Supplementary Figure 2**), weekly time (*p* = 0.636, **Supplementary Figure 3**), session duration (*p* = 0.297, **Supplementary Figure 4**), age (*p* = 0.213, **Supplementary Figure 5**), or disease duration (*p* = 0.569, **Supplementary Figure 6**) and FMD.

### 3.6 Publication bias

**Figure 8:**
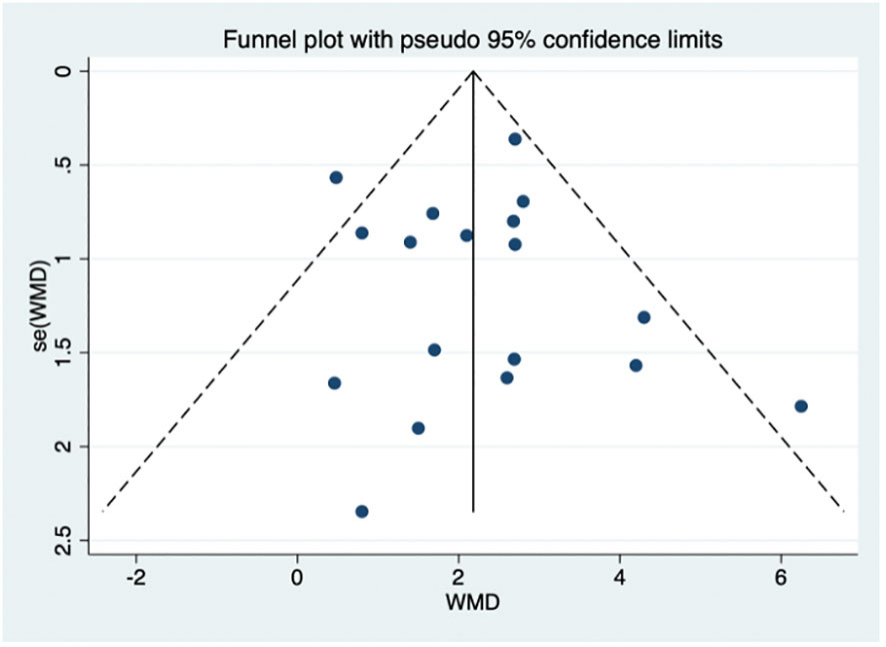
Funnel plot.

Possible publication bias was evaluated by the funnel plot (**Figure 8**). Visual inspection of the funnel plot suggested the absence of funnel plot asymmetry. Based on the results of egger’s test, small sample size studies were insufficient to affect the final results (*p* = 0.775, **Supplementary Table 4**).

### 3.7 Sensitivity analysis

Sensitivity analysis showed that there is no change in the direction or level of compatibility of the overall effect of exercise on FMD in T2DM patients when any of the included studies are omitted (**Supplementary Figure 7**).

## 4 Discussion

### 4.1 Effects of exercise on FMD in T2DM patients

In this study, we aimed to investigate the effect of exercise on FMD in T2DM patients and the optimal type, frequency, session duration, and weekly time of exercise for T2DM patients, and a total of 13 studies containing data from 523 patients were included. Our results showed that exercise significantly improved FMD in T2DM patients, which was consistent with the results of previous studies (50–52). In terms of WMD, exercise improved FMD by 2.18% in T2DM patients, which has significant clinical implications for individuals with T2DM. A previous meta-analysis showed that for every 1% increase in FMD, the risk of cardiovascular events was expected to decreased by 13% (53). Meanwhile, we noted the inclusion of studies combining exercise and dietary interventions in previous meta-analyses (50, 51), which may be due to the limited number of studies on the effect of exercise interventions alone on endothelial function in T2DM patients. Notably, our meta-analysis avoided this limitation by excluding studies combining exercise and dietary interventions (54–56), and none of the 13 included studies involved dietary interventions. This is because dietary interventions may have a confounding effect with exercise, thus masking the true efficacy of exercise in T2DM patients (57, 58).

Although the exact mechanisms remain incompletely elucidated, it can be hypothesized that the benefits of exercise on endothelial function can be amplified through the following mechanisms. First of all, shear stress plays a central role in regulating the inflammatory response of the vascular endothelium and the pathogenesis of atherosclerosis (59). Several studies have shown that exercise leads to an increase in blood flow, which in turn increases the shear stress of blood flow (60, 61), suggesting that the vascular endothelium is induced to synthesize more NO synthesis and increase the bioavailability of NO (62, 63). Secondly, both oxidative stress and inflammation are among the risk factors for vascular endothelial diseases (64, 65), and both are initiating factors for endothelial dysfunction. However, exercise has been shown to have anti-inflammatory properties and to reduce oxidative stress as a non-pharmacological intervention (66, 67). On the one hand, exercise reduces low-grade inflammation biomarkers and endothelial dysfunction biomarkers in plasma (68). In addition, exercise also affects oxidative stress by increasing the availability of antioxidant enzymes, thus improving endothelial function (62, 69). Furthermore, endothelial progenitor cells (EPCs) may also server as biomarkers of cardiovascular function (70), and Ribeiro et al. (71) showed that exercise increased the number and differentiation capacity of EPCs, which may contribute to vascular regeneration and angiogenesis. Thus, an increased in the number of EPCs may positively affect endothelial function (72, 73).

### 4.2 Subgroup analysis

Subgroup analysis of different types of intervention showed that HIIT, aerobic exercise, resistance exercise, and multi-component training were all effective in improving FMD in T2DM patients, with HIIT being the most effective intervention type, although aerobic exercise is widely used to improve chronic disease. This may be due to the fact that intensity is an important factor in FMD (28), and HIIT tends to be higher in intensity compared to aerobic exercise or other interventions. Thijssen et al. (74) showed that vascular blood flow and shear stress improved with increasing exercise intensity. Elevated vascular shear stress due to HIIT would lead to potassium channel activation and increased Ca^2+^ entering the vascular endothelium. Elevated intracellular Ca^2+^ concentration triggers activation of endothelial nitric oxide synthase (eNOS) (75, 76). In addition, HIIT may also lead to decreased catecholamine levels and α-adrenoceptor density (77, 78). Furthermore, adropin, as a regulator of eNOS synthase and NO release, has been implicated as a potential factor affecting endothelial function. A previous study showed that elevated adropin levels increased eNOS mRNA expression (79), indicating that elevated adropin may contribute to the reduction of exercise-induced atherosclerosis (80). Thus, elevated adropin may be considered a marker of improved endothelial function (24). Although the mechanism by which exercise leads to elevated adropin levels is unknown, it was observed in one study that a 12-week HIIT intervention significantly increased adropin levels in T2DM patients (24). Meanwhile, in another clinical trial using HIIT and MICT as interventions, a greater increase in adropin was observed in the HIIT group than in the MICT group (26). Moreover, a recent study (73) has shown that HIIT is superior to MICT in mobilizing circulating EPCs. All of these mechanisms appear to lead to greater NO production and increased NO bioavailability, thus well explaining the further improvement in FMD. Moreover, our result was consistent with a meta-analysis conducted by Ramos et al. (81), showing that HIIT was more effective in improving FMD compared to MICT.

Regarding intervention frequency, our subgroup analysis showed that interventions performed more than 3 times per week had a greater improvement in FMD compared to interventions performed up to 3 times per week, which was in agreement with a previous study (30), showing that high-frequency interventions are more beneficial than low-frequency interventions for endothelial function in T2DM patients. This hypothesis is also supported by a meta-analysis conducted by Fuertes-Kenneally et al. (82), showing that a higher frequency of intervention per week was associated with a better effect on endothelial function improvement. However, we believe that the frequency of intervention may be influenced by other factors, such as session duration and weekly time.

It is reported that the effects of exercise on health have a dose-response relationship, and that it is not more exercise that is beneficial, but rather the appropriate load that determines the health benefits of exercise (83). Several studies have found that engaging in extraordinarily prolonged exercise does not seem to provide corresponding benefits to the body and can even trigger negative effects on cardiac function (84–86). The benefits of exercise on endothelial function seem to apply here as well. Our subgroup analysis showed greater improvements in FMD in T2DM patients with exercise conducted less than 60 min compared to 60 min or more per session. It has been shown that T2DM patients typically have lower exercise tolerance (39), which may make it difficult for them to perform prolonged exercise during each session. Therefore, it can be concluded that a longer exercise duration does not contribute to more improvement in T2DM patients, and that a single session of less than 60 min may be more favorable for adherence to exercise and associated health benefits in T2DM patients.

However, our previous study found that the use of frequency and session duration alone did not exclude the influence of other variables (49). Therefore, we considered introducing weekly time to provide new ideas for exercise prescription. The weekly time was calculated based on the frequency and session duration. The World Health Organization (WHO) recommends that people perform 150-300 min of moderate-intensity aerobic exercise, 75-150 minutes of vigorous-intensity aerobic exercise, or an equal combination of moderate- and vigorous-intensity each week (87). Our subgroup analysis showed that a shorter weekly time (≤ 180 min *vs.* > 180 min) were associated with a larger improvement in FMD, which may also be related to the exercise tolerance of T2DM patients, for which more than 180 min per week does not seem to provide additional physical benefits.

### 4.3 Strengths and limitations of this systematic review

In this systematic review and meta-analysis, we included studies on the effect of exercise interventions alone on FMD in T2DM patients, and excluded studies where exercise was combined with dietary interventions, which can better reflect the effect of exercise interventions. Our findings provide an optimal combination of exercise modalities for T2DM patients. Clinically, T2DM patients can improve endothelial function by engaging in exercise 3 times per week for less than 60 min each time, especially HIIT, to achieve the goal of 180 min of exercise per week.

However, the present study has some limitations that should be noted. Although previous studies have found that improvement in FMD in T2DM patients decreases as the duration of intervention increases, it was not possible to investigate the effect of duration on the degree of improvement in FMD because the duration of the interventions in the included studies was generally focused on 12 weeks. In addition, although our study found that HIIT is the most effective intervention type for improving FMD in T2DM patients. However, due to the limited number of studies using HIIT in T2DM patients, we were unable to examine the optimal design of HIIT interventions. Finally, the studies we included contained aerobic exercise, HIIT, resistance exercise, and multicomponent exercise, and we were unable to standardize the intensity of exercise, so we were unable to explore the effect of intensity on FMD in T2DM patients.

## 5 Conclusion

In this meta-analysis, exercise had beneficial effect in improving FMD in T2DM patients, with HIIT being the most effective intervention type. To improve endothelial function, this meta-analysis provides clinicians with evidence to recommended that T2DM patients participate in exercise, especially HIIT, more than 3 times per week for less than 60 min, with a target of 180 min per week being reached by increasing the frequency of exercise.

## Data availability statement

The original contributions presented in the study are included in the article/**Supplementary Material**. Further inquiries can be directed to the corresponding author.

## Author contributions

BQ: Conceptualization, Data curation, Formal analysis, Investigation, Methodology, Software, Validation, Visualization, Writing – original draft, Writing – review & editing. YZ: Data curation, Formal analysis, Investigation, Methodology, Software, Validation, Visualization, Writing – original draft. XT: Data curation, Formal analysis, Writing – review & editing. XH: Data curation, Formal analysis, Writing – review & editing. LD: Data curation, Formal analysis, Writing – review & editing. YL: Data curation, Formal analysis, Writing – review & editing. LY: Conceptualization, Data curation, Funding acquisition, Methodology, Project administration, Resources, Supervision, Writing – review & editing.
