# Supplementary material for: The effect of exercise on flow-mediated dilation in people with type 2 diabetes mellitus: a systematic review and meta-analysis of randomized controlled trials
Source: Front Endocrinol (Lausanne). 2024 Mar 26;15:1347399. doi: 10.3389/fendo.2024.1347399 (PMC11002232; doi:10.3389/fendo.2024.1347399)
Supplement: Supplementary file 1 [file DataSheet_1.pdf]

Supplementary Material

The effect of exercise on flow-mediated dilation in people with type 2 diabetes mellitus: a systematic review and meta-analysis of randomized controlled trials

Bopeng Qiu, Yilun Zhou, Xifeng Tao, Xiao Hou, Liwen Du, Yuanyuan Lv, Laikang Yu*

*** Correspondence:** Laikang Yu, yulaikang@126.com

1. Supplementary Figures

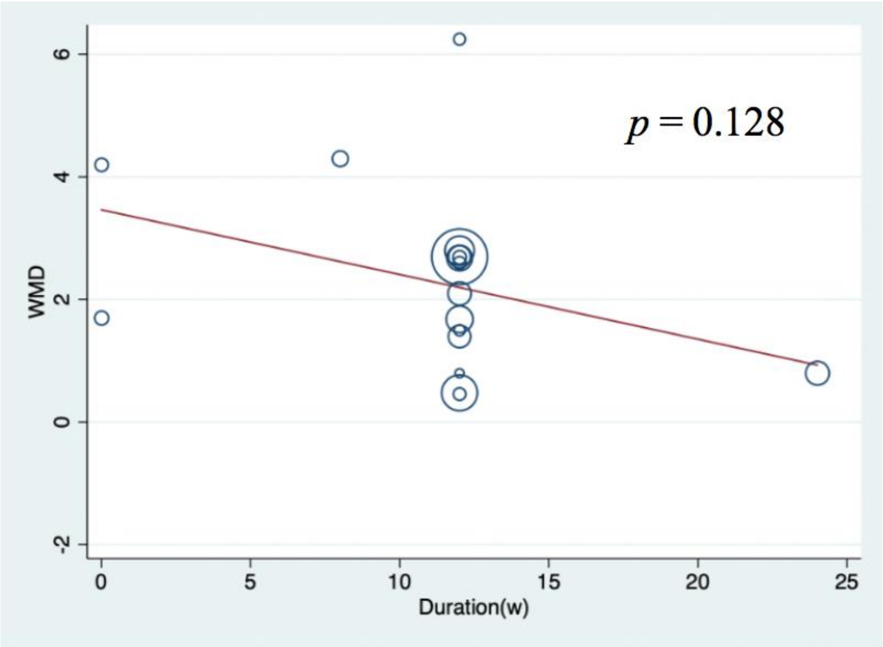

Supplementary Figure 1. Meta-regression analysis result of intervention duration.

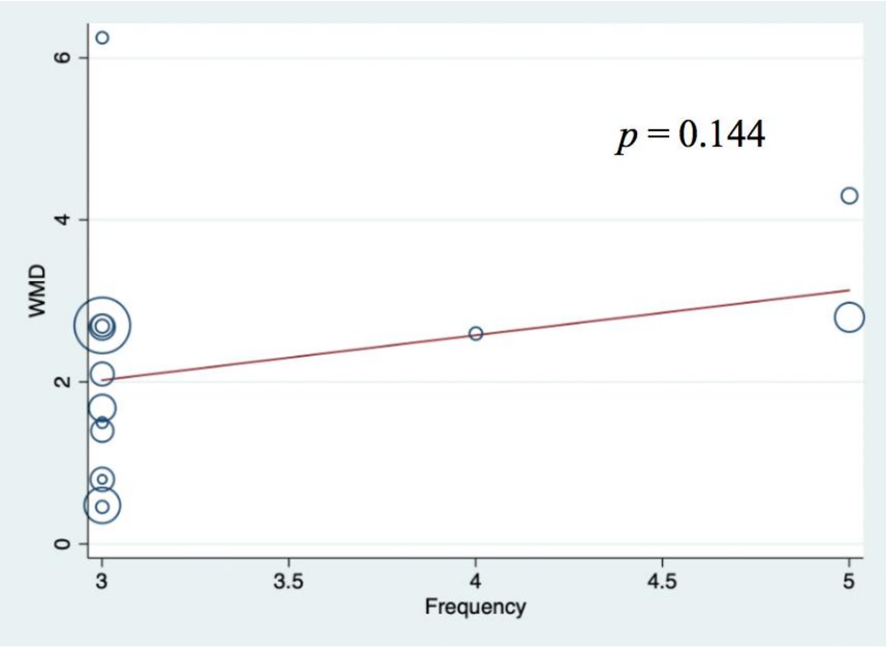

Supplementary Figure 2. Meta-regression analysis result of frequency.

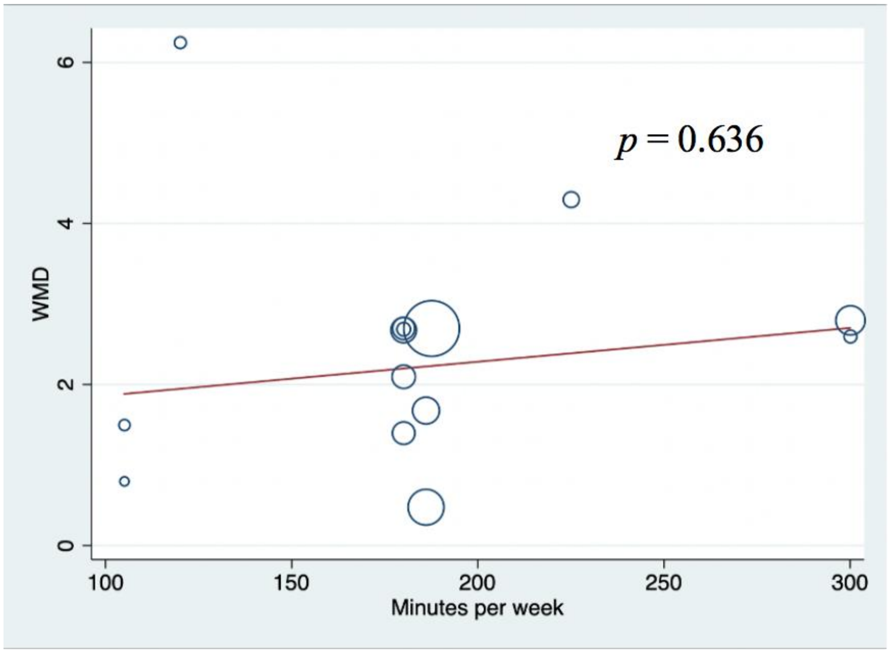

Supplement Figure 3. Meta-regression analysis result of weekly time.

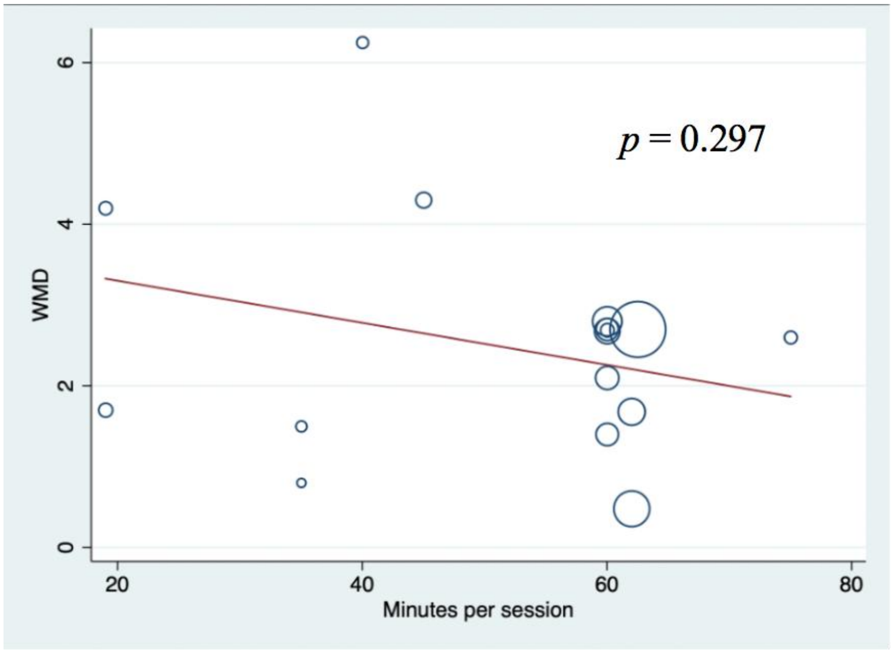

Supplement Figure 4. Meta-regression analysis result of session duration.

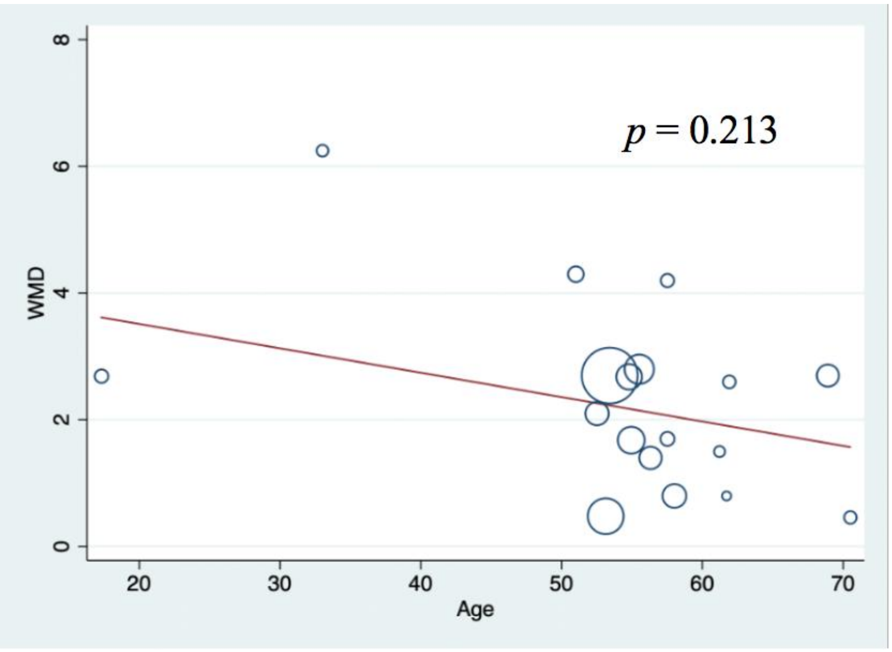

Supplement Figure 5. Meta-regression analysis result of age.

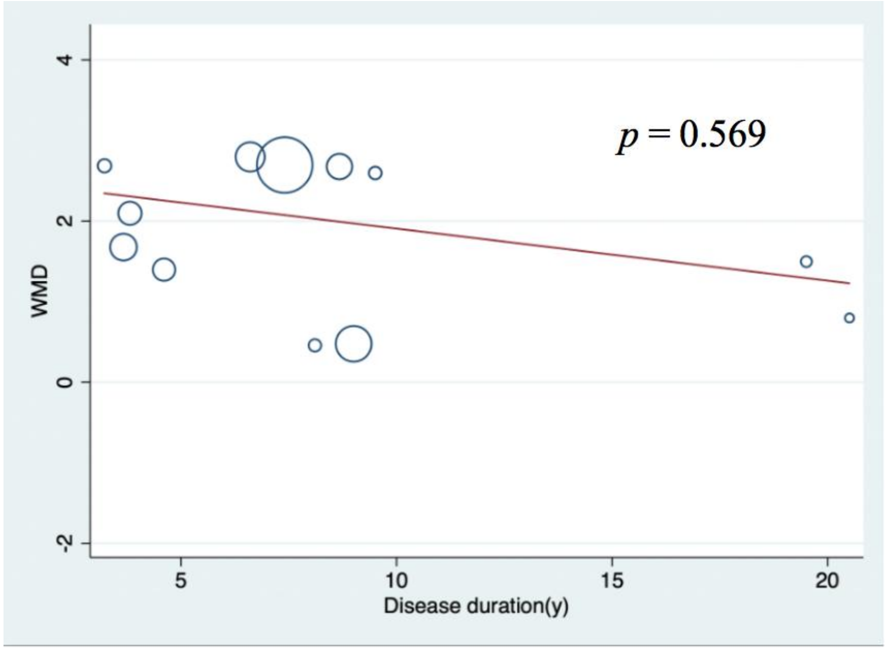

Supplement Figure 6. Meta-regression analysis result of disease duration.

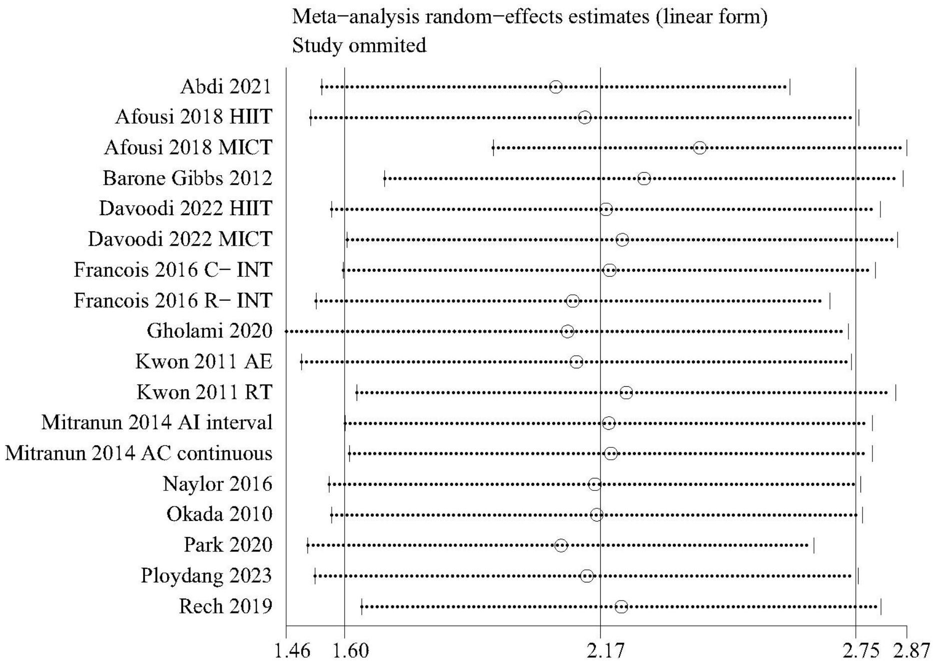

Supplement Figure 7. Sensitivity analysis result.

2. Supplementary Tables

Supplementary Table 1. Search terms for exercise, Type 2 diabetes mellitus, and endothelial function

**Exercise search terms combined with 'OR'**
Physical exercise programs; Physical Therapy Modalities; Physical Therapy Modalities; Modalities, Physical Therapy; Modality, Physical Therapy; Physical Therapy Modality; Physiotherapy; Physiotherapies; Physical Therapy Techniques; Physical Therapy Technique; Techniques, Physical Therapy; Exercise; Exercise Movement Techniques; Exercise Movement Techniques; Movement Techniques, Exercise; Exercise Movement Technics; Exercise Therapy; Exercise Therapy; Therapy, Exercise; Exercise Therapies; Therapies, Exercise; Exercise, Physical; Exercises, Physical; Physical Exercise; Physical Exercises; Exercise, Isometric; Exercises, Isometric; Isometric Exercises; Isometric Exercise; Exercise, Aerobic; Aerobic Exercises; Aerobic Exercise; Resistance Training; Resistance Training; Training, Resistance; Strength Training; Training, Strength; Weight-Lifting Strengthening Program; Strengthening Program, Weight-Lifting; Strengthening Programs, Weight-Lifting; Weight Lifting Strengthening Program; Weight-Lifting Strengthening Programs; Weight-Lifting Exercise Program; Exercise Program, Weight-Lifting; Exercise Programs, Weight-Lifting; Weight Lifting Exercise Program; Weight-Lifting Exercise Programs; Weight-Bearing Strengthening Program; Strengthening Program, Weight-Bearing; Strengthening Programs, Weight-Bearing; Weight Bearing Strengthening Program; Weight-Bearing Strengthening Programs; Weight-Bearing Exercise Program; Exercise Program, Weight-Bearing; Exercise Programs, Weight-Bearing; Weight Bearing Exercise Program; Weight-Bearing Exercise Programs; Stretching; Muscle Stretching Exercises; Exercise, Muscle Stretching; Muscle Stretching Exercise; Static Stretching; Stretching, Static; Active Stretching; Stretching, Active; Static-Active Stretching; Static Active Stretching; Stretching, Static-Active; Isometric Stretching; Stretching, Isometric; Ballistic Stretching; Stretching, Ballistic; Dynamic Stretching; Stretching, Dynamic; Proprioceptive Neuromuscular Facilitation (PNF) Stretching; PNF Stretching; PNF Stretchings; Stretching, PNF; PNF Stretching Exercise; Exercise, PNF Stretching; PNF Stretching Exercises; Stretching Exercise, PNF; Proprioceptive Neuromuscular Facilitation; Neuromuscular Facilitation, Proprioceptive; Proprioceptive Neuromuscular Facilitations; Passive Stretching; Stretching, Passive; Relaxed Stretching; Stretching, Relaxed; Static-Passive Stretching; Static Passive Stretching; Stretching, Static-Passive; Yoga; Flexibility
**Type 2 diabetes mellitus search terms combined with 'OR'**
Diabetes Mellitus, Type 2; Diabetes Mellitus, Noninsulin-Dependent; Diabetes Mellitus, Ketosis-Resistant; Diabetes Mellitus, Ketosis Resistant; Ketosis-Resistant Diabetes Mellitus; Diabetes Mellitus, Non Insulin Dependent; Diabetes Mellitus, Non-Insulin-Dependent; Non-Insulin-Dependent Diabetes Mellitus; Diabetes Mellitus, Stable; Stable Diabetes Mellitus; Diabetes Mellitus, Type II; NIDDM; Diabetes Mellitus, Noninsulin Dependent; Diabetes Mellitus, Maturity-Onset; Diabetes Mellitus, Maturity Onset; Maturity-Onset Diabetes Mellitus; Maturity Onset Diabetes Mellitus; MODY; Diabetes Mellitus, Slow-Onset; Diabetes Mellitus, Slow Onset; Slow-Onset Diabetes Mellitus; Type 2 Diabetes Mellitus; Noninsulin-Dependent Diabetes Mellitus; Noninsulin Dependent Diabetes Mellitus; Maturity-Onset Diabetes; Diabetes, Maturity-Onset; Maturity Onset Diabetes; Type 2 Diabetes; Diabetes, Type 2; Diabetes Mellitus, Adult-

Onset; Adult-Onset Diabetes Mellitus; Diabetes Mellitus, Adult Onset
**Endothelial function search terms combined with 'OR'**
Endothelium; vascular function; endothelial; flow-mediated dilation; flow mediated dilation; endothelial function; FMD; artery blood flow

Supplementary Table 2. Characteristics of the studies included in the systematic review

Study	Sample Size (M/F)	Mean Age(y)	Disease Duration(y)	Intervention	Exercise Protocol	Minutes Per session (min)	Frequency	Minutes Per week (min)	Duration	Results on FMD
Abdi et al., 2021	INT:15 (0/15)<br>CON:15 (0/15)	20 to 44	Over two years	HIIT	Four 4-min running intervals at 85 - 95% of HR_{max} separated by 3-min intervals at 50-60% of HR_{max}.	40	3	120	12 weeks	Increase
Afousi et al., 2018	HIIT: 18(9/9)<br>MICT: 17(10/7)<br>CON:17(9/8)	HIIT: 54.78 ± 6.19<br>MICT: 53.12 ± 4.84<br>CON: 54.24 ± 5.61	HIIT: 8.67 ± 2.40<br>MICT: 9 ± 2.39<br>CON: 8.47 ± 2.12	HIIT; MICT	HIIT: consisted of 12 intervals (1.5 min) at 85%-90% HR_{max} separated by 2 min at 55%- 60% HR_{max};<br><br>MICT: cycled for 42 min at 70% HR_{max}.	HIIT: 60<br>MICT: 62	3	HIIT: 180<br>MICT: 186	12 weeks	Increase
Barone Gibbs et al., 2012	INT: 46<br>CON:63(37/26)	INT: 58 ± 5<br>CON:56 ± 6	NR	AE combined with RE	Aerobic exercise at 60-90% HR_{max}, along with 7 weight training exercises with two sets of 12-15 reps at 50% of 1-repetition maximum for each session.	AE: 55 to 60<br>RT: NR	3	AE: 165 to 180	24 weeks	No change
Davoodi et al., 2022	HIIT: 16(9/7)<br>MICT: 16(10/6)<br>CON: 15(8/7)	HIIT: 52.50 ± 7.22<br>MICT: 54.93 ± 5.56<br>CON: 53.66 ± 6.05	HIIT: 3.81 ± 1.47<br>MICT: 3.66 ± 1.39<br>CON: 4.86 ± 1.76	HIIT; MICT	HIIT: consisted of 12 intervals (1.5 min) at 85%-90% HR_{max} separated by 2 min at 55%- 60% HR_{max};<br><br>MICT: cycled for 42	HIIT: 60<br>MICT: 62	3	HIIT: 180<br>MICT: 186	12 weeks	Increase

					min at 70% HR_{max}.					
Francois et al., 2016	HIIT: 12(6/6)<br>RE: 12(6/6)<br>CON: 12(6/6)	57.5 ± 5.0	NR	HIIT; RE	HIIT: seven 1-min intervals on the cycle ergometer at 85% W_{peak}, alternated with 1-min recovery at 15% W_{peak};<br><br>RE: seven 1-minute leg resistance intervals, for each 1-min interval participants completed as many reps as possible of each exercise, alternated with 1-min recovery.	19	NA	NA	NA	RT Increase
Gholami et al., 2020	INT: 16(16/0)<br>CON: 15(15/0)	INT: 53.4 ± 9.1<br>CON: 52.2 ± 8.5	INT: 7.4 ± 5.1<br>CON: 8.4 ± 4.5	HIIT	Started with 50% of HRR for 20 min at familiarization sessions and reached 70% of HRR for 45 min during last weeks.	55 to 70	3	165 to 210	12 weeks	Increase
Kwon et al., 2011	AE: 13(0/13)<br>RE: 12(0/12)<br>CON: 15(0/15)	AE:55.5 ± 8.6<br>RT: 56.3 ± 6.1<br>CON: 58.9 ± 5.7	AE: 6.6 ± 6.7<br>RE: 4.6 ± 2.7<br>CON: 4.9 ± 4.7	AE; RE	AE: walking at moderate intensity, intensity is controlled at 3.6 to 6.0 METs;<br><br>RE: resistance bands exercise at gradually increased intensities by up to 40-50%, three sets of 10-15 exercises for each session.	60	AE: 5<br>RT: 3	AE: 300<br>RT: 180	12 weeks	AE Increase

Mitranun et al., 2014	AI: 14(5/9)<br>AC: 14(5/9)<br>CON: 15(5/10)	AI: 61.2 ± 10.48<br>AC: 61.7 ± 10.10<br>CON: 60.9 ± 9.30	AI: 19.5 ± 1.50<br>AC: 20.5 ± 1.50<br>CON: 21.1 ± 2.32	AE	AI: walking or running consisted of 4-6 intervals of 1 min exercise at 80-85% VO_{2peak} with a 4 min exercise at 50-60% VO_2peak;<br><br>AC: walking or running at 60-65% VO_2peak.	Weeks 1 to 6: 30<br>Weeks 7 to 12: 40	3	90 to 120	12 weeks	Increase
Naylor et al., 2016	INT: 8(2/6)<br>CON:5(1/4)	INT: 17.3 ± 2.26<br>CON: 15.3 ± 1.79	INT: 38.6 ± 19.52<br>CON: 17.2 ± 16.77	AE combined with RE	Combined aerobic exercise at 65-85% HR_{max} and resistance training at 55-70% MVC, with training volume gradually increased.	60	3	180	12 weeks	Increase
Okada et al., 2010	INT: 21(10/11)<br>CON:17(11/6)	INT: 61.9 ± 8.6<br>CON: 64.5 ± 5.9	INT: 9.5 ± 8.1<br>CON: 10.8 ± 7.4	AE combined with RE	Each session consisted of a 10-minute warm-up, then aerobic dance, stationary bike ride, and resistance training were all 20 minutes each ending with a 5 min cool-down. Training heart rate was determined using the Kavonen equation.	75	3 ~ 5	225 to 375	12 weeks	Increase
Park et al., 2020	INT: 10(0/10)<br>CON: 10(0/10)	T2DM: 51 ± 10.20	NR	AE	The walking workload was individually adjusted throughout the intervention to maintain 60% HRR.	45	5	225	8 weeks	Increase

Ployda ng et al., 2023	INT: 16(5/11)<br>CON: 17(7/10)	INT: 68.9 ± 3.7<br>CON: 69.2 ± 5.3	NR	AE	Each exercise bout was 40 min in duration at 40%-50% HRR for the first 6 weeks and 50%-60% HRR for the last 6 weeks.	60	3	180	12 weeks	Increase
Rech et al., 2019	INT: 17(10/7)<br>CON: 21(10/11)	INT: 70.5 ± 7.4<br>CON: 68 ± 6.5	INT: 8.1 ± 5.8<br>CON: 11.2 ± 7.5	RE	The RE program featured functional exercises like partial squats and bench stepping, intensity monitored by the OMNI scale. Resistance exercises progressed from 12 to 10 reps, avoiding muscle failure, with 2-3 sets per exercise.	NR	3	NR	12 weeks	No change

Abbreviations: INT, Intervention group; CON, Non-intervention control group; AE, Aerobic exercise; AI, Interval aerobic exercise training; AC, Continuous aerobic exercise training; RE, Resistance exercise; HIIT, High-intensity interval training; HRR, Heart rate reserve; MICT, Moderate intensity continuous training; MVC, Maximum voluntary contraction, NR, Not reported; NA, Not Applicable; VO_2peak, peak oxygen uptake.

Supplement Table 3. Methodological assessment of randomized controlled trials included in the systematic review using the PEDro scale

Study	1	2	3	4	5	6	7	8	9	10	11	Score
Abdi et al., 2021	Y	1	0	1	1	1	1	0	0	1	1	7/10
Afousi et al., 2018	Y	1	0	1	1	1	1	1	0	1	1	8/10
Barone Gibbs et al., 2012	Y	1	0	1	1	1	1	1	0	1	1	8/10
Davoodi et al., 2022	Y	1	0	1	1	1	1	1	0	1	1	8/10
Francois et al., 2016	Y	1	0	0	1	1	1	0	0	1	1	6/10
Gholami et al., 2020	Y	1	1	0	1	1	1	1	0	1	1	8/10
Kwon et al., 2011	Y	1	0	1	1	1	1	1	1	1	1	9/10
Mitranun et al., 2014	Y	1	0	0	1	1	1	1	0	1	1	7/10
Naylor et al., 2016	Y	1	0	1	1	1	1	1	0	1	1	8/10
Okada et al., 2010	Y	1	0	1	1	1	1	1	1	0	1	8/10
Park et al., 2020	Y	1	0	0	1	1	1	0	0	1	1	6/10
Ploydang et al., 2023	Y	1	0	1	1	1	1	0	0	1	1	7/10
Rech et al., 2019	Y	1	1	1	1	1	1	0	0	1	1	8/10

1 = eligibility criteria; 2 = random allocation; 3 = concealed allocation; 4 = baseline comparability; 5 = blind subjects; 6 = blind therapists; 7 = blind assessors; 8 = adequate follow-up; 9 = intention-to-treat analysis; 10 = between-group comparisons; 11 = point estimates and variability. The total score represents the score of the PEDro scale. Item 1 was not scored. Y: yes

Supplement Table 4. Results of egger's test

Coef.	SE	95% CI	t	*p*	Number of trials
0.189	0.648	−1.184 to 1.561	0.29	0.775	18
